# Supplementary material for: Podocyte-specific KLF6 primes proximal tubule CaMK1D signaling to attenuate diabetic kidney disease
Source: Nat Commun. 2024 Sep 13;15:8038. doi: 10.1038/s41467-024-52306-5 (PMC11399446; doi:10.1038/s41467-024-52306-5)
Supplement: Supplementary file 1 — Supplementary Information [file 41467_2024_52306_MOESM1_ESM.pdf]

Supplementary Table 1. Blood glucose, kidney weight, and body weight

	SHAM + VEH (non-diabetic)		UNx + STZ (diabetic)	
**Genotype**	***NPHS2-rtTA***	***KLF6^{PODTA}***	***NPHS2-rtTA***	***KLF6^{PODTA}***
Body weight (g) at 10 weeks	28.2 ± 0.6	28 ± 0.5	27.6 ± 0.5	28.7 ± 0.4
Body weight (g) at 20 weeks	30.7 ± 0.4	31.2 ± 0.7	25.9 ± 0.5****	27.3 ± 0.5**
Change in body weight (g)	2.8 ± 0.2	3.6 ± 0.3	-1.9 ± 0.4***	-1.69 ± 0.4**
Kidney weight (g)	0.21 ± 0.02	0.19 ± 0.01	0.42 ± 0.02 ****	0.44 ± 0.02 **
% Kidney weight/Body weight	0.69 ± 0.04	0.61 ± 0.04	1.64 ± 0.08***	1.59 ± 0.06**
Blood glucose before UNx or SHAM UNx (mg/dl)	140 ± 7.0	146 ± 6.4	145 ± 6.6	151 ± 4.8
Blood glucose post STZ or VEH treatment (mg/dl)	204 ± 26	161 ± 22	>600 ± 0.0 ***	>600 ± 0.0 **

Values are mean±SEM; SHAM + VEH (n=5 for *NPHS2-rtTA* and 4 for *KLF6^{PODTA}*), UNx +STZ (n=18 for *NPHS2-rtTA* and 24 for *KLF6^{PODTA}*) ; body weight at 20 weeks, Mann Whitney t-test (two-sided), p=<0.0001 for *NPHS2-rtTA* (SHAM + VEH vs UNx + STZ) and p=0.0098 for *KLF6^{PODTA}* (SHAM + VEH vs UNx + STZ); change in body weight, Mann Whitney t-test(two-sided), p=0.0005 for *NPHS2-rtTA* (SHAM + VEH vs UNx + STZ), p=0.0018 for *KLF6^{PODTA}* (SHAM + VEH vs UNx + STZ); kidney weight, Mann Whitney t-test (two sided), p=<0.0001 for for *NPHS2-rtTA* (SHAM + VEH vs UNx + STZ), p=0.0021 for *KLF6^{PODTA}* (SHAM + VEH vs UNx + STZ); % Kidney weight/body weight, MannWhitney t-test(two-sided), p=0.0001 for for *NPHS2-rtTA* (SHAM + VEH vs UNx + STZ), p=0.0021 for *KLF6^{PODTA}* (SHAM + VEH vs UNx + STZ); blood glucose post STZ or VEH treatment, Mann Whitney t-test (two sided), p=0.0002 for *NPHS2-rtTA* (SHAM + VEH vs UNx + STZ), p=0.0012 for *KLF6^{PODTA}* (SHAM + VEH vs UNx + STZ) **p<0.01, ***p<0.001, ****p<0.0001 versus the sham-treated *NPHS2-rtTA* mice; Kruskal-Wallis test. UNx (uninephrectomy); STZ (streptozotocin); VEH (vehicle).

Supplementary Table 2. Histopathological scoring

	SHAM + VEH (non-diabetic)		UNx + STZ (diabetic)	
**Genotype**	***NPHS2-rtTA***	***KLF6^{PODTA}***	***NPHS2-rtTA***	***KLF6^{PODTA}***
**Sclerotic glomeruli**	0.0	0.0	55 ± 7*	17.3 ± 2.6*#
**Tubular injury score**	0.0	0.0	2 ± 0.6	0.0
**Interstitial fibrosis score**	0.0	0.0	0.7 ± 0.3	0.0
**Inflammation score**	0.0	0.0	0.7 ± 0.3	0.3 ± 0.3

Values are mean±SEM; n=3. Sclerotic glomeruli are scored as the percentage of glomeruli that are sclerotic. Tubular injury score, interstitial fibrosis score and inflammation scores are scored from 0 to 3, 0 = none, 1 = mild (5-25%), 2 = moderate (>25-<50%) and 3 = severe (>50%); p=0.0247 for *KLF6^{PODTA} vs NPHS2-rtTA* (UNx + STZ), p=0.0170 for *NPHS2-rtTA* (SHAM + VEH vs UNx + STZ), p=0.0218 for *KLF6^{PODTA}* (SHAM + VEH vs UNx + STZ); *p<0.05 versus respective SHAM+VEH treated mice; #p<0.05 versus UNx + STZ *NPHS2-rtTA* mice; Welch's t-test, two-sided.

Supplementary Table 3. Primer sequences for genotyping

**Gene**	**Forward Primer**	**Reverse Primer**
TRE-hKLF6	GCTGCCGTCTCTGGAGGAGT	CAGGGCTCGCTCTGGAGGTA
NPHS2-rtTA	GAACAACGCCAAGTCATTCCG	TACGCAGCCCAGTGTAAAGTGG
TRE-GFP	AAGTTCATCTGCACCACCG	TCCTTGAAGAAGATGGTGCG

Supplementary Table 4. Primer sequences for real-time PCR

**Gene**	**Forward Primer**	**Reverse Primer**
Mouse-*Klf6*	ACACGTAGCAGGGCTCACTC	CACGAAACGGGCTACTTCTC
Human-*KLF6*	GCTGCCGTCTCTGGAGGAGT	CAGGGCTCGCTCTGGAGGTA
Mouse-*Camk1d*	CCAAGCATAGTCCAGGGCAA	AGAGCTGAAGGGAACCGTTG
Human-*CAMK1D*	GAGAGCAGCTCCTCCTGGAA	AGGCCCCGGTTCCGA
Mouse-*Actb*	GTTCCGATGCCCTGAGGCTCTT	CGTCACACTTCATGATGGAATTGA
Human-*ACTB*	AGAGCTACGAGCTGCCTGAC	AGCACTGTGTTGGCGTACAG
Human-*APOJ*	TGCGGATGAAGGACCAGTGTGA	TTTCCTGGTCAACCTCTCAGCG

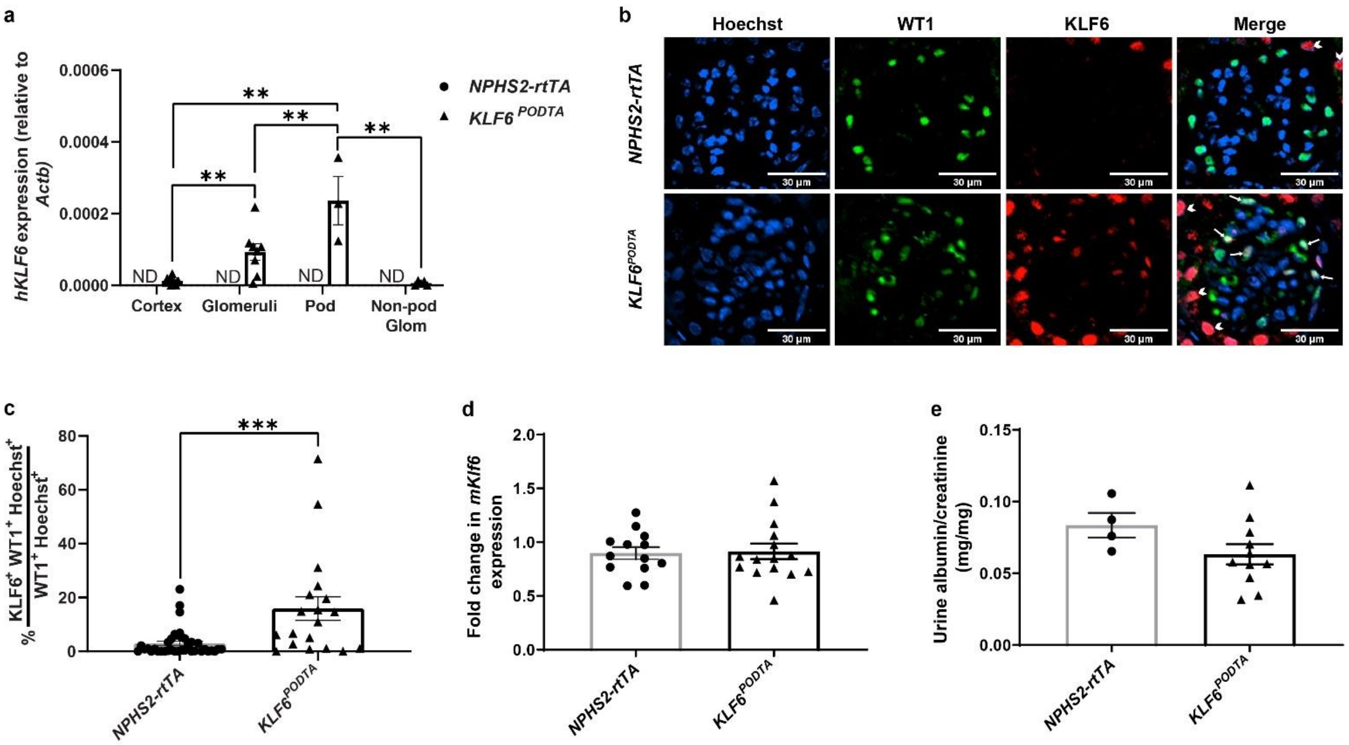

Supplementary Fig. 1. Generation of *KLF6*^{PODTA} mice. (a) Human *KLF6* expression (relative to mouse *Actb*) in kidney cortex, glomerular, podocyte, and non-podocyte glomerular fractions from *NPHS2-rtTA* and *KLF6*^{PODTA} mice (n=10 mice for cortex, 9 mice for glomeruli, 3 mice for podocyte and, 5 mice for non-podocyte, p=0.0038 for cortex vs glomeruli, p=0.0060 for glomeruli vs podocytes, p=0.0011 for cortex vs podocytes, p=0.0016 for podocytes vs non-podocytes, **p<0.01; Kruskal-Wallis test with Dunn's post-test, data presented as mean±SEM). **(b-c)** Representative images of KLF6, WT1, and Hoechst immunostaining in the glomerulus and quantification of podocyte-specific expression of KLF6. Arrows indicate KLF6 and WT1 colocalization; arrowheads indicate tubular KLF6 staining (n=6 glomeruli/mouse, n=3 mice/group, p=0.003, ***p<0.001; Welch's t-test, data presented as mean±SEM). **(d)** mouse *Klf6* expression in *KLF6*^{PODTA} relative to *NPHS2-rtTA* mice (n=13 *NPHS2-rtTA* mice and 15 *KLF6*^{PODTA} mice; data presented as mean±SEM). **(e)** Urine albumin/creatinine ratio (mg/mg) in *KLF6*^{PODTA} and *NPHS2-rtTA* mice (n=4, *NPHS2-rtTA* mice and 11 mice *KLF6*^{PODTA} mice, data presented as mean±SEM). Source data are provided as a Source Data file.

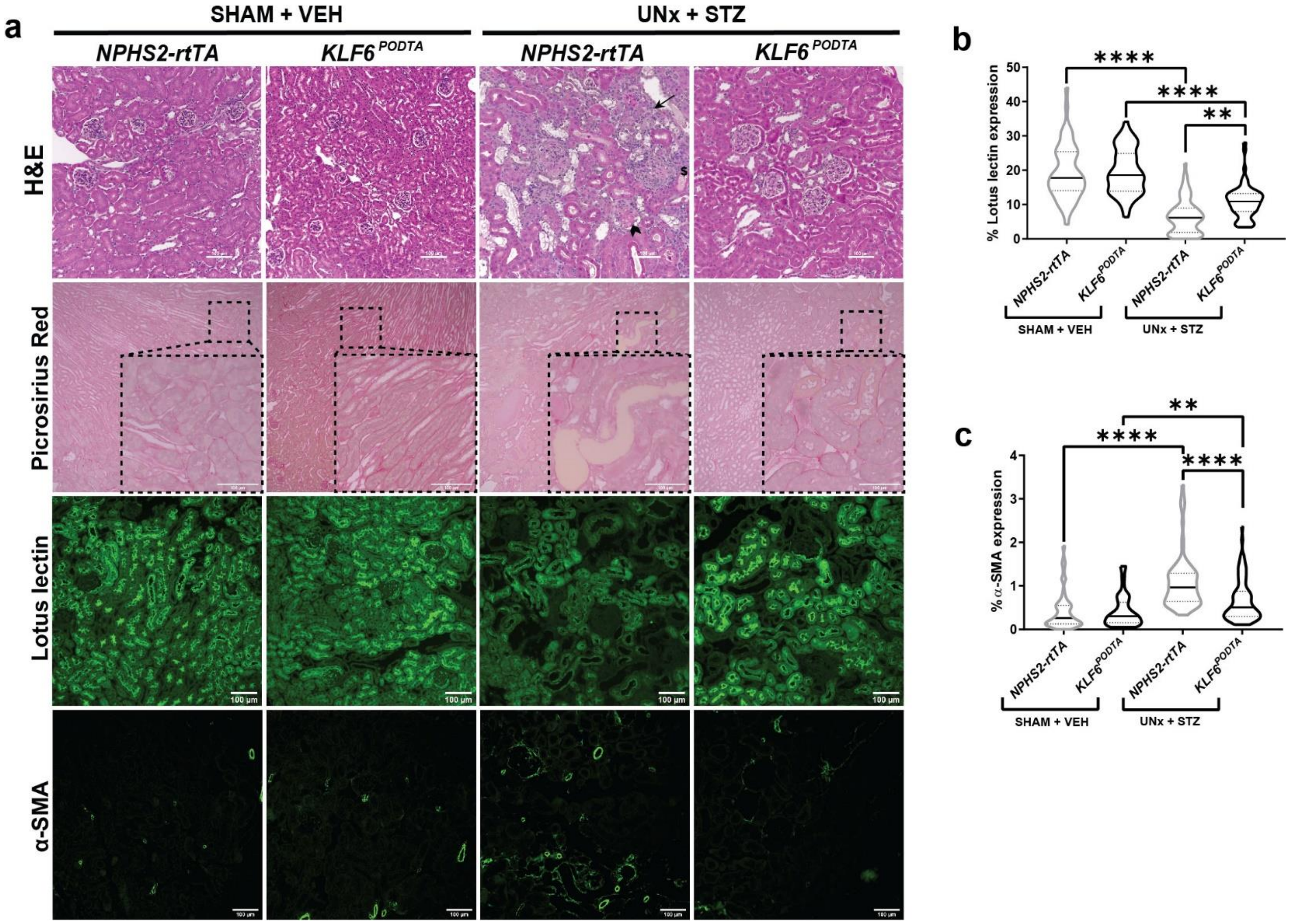

Supplementary Fig. 2. *KLF6^{PODTA}* mice exhibit less tubular injury and interstitial fibrosis than *NPHS2-rtTA* mice under diabetic conditions. (a) Representative images of H&E (20x), picrosirius red staining (with inset), lotus lectin staining, and alpha smooth muscle actin (α-SMA) immunostaining. Black arrowheads indicate sclerotic glomeruli; $ indicates protein casts; and black arrows indicate areas of interstitial inflammation. Quantification of **(b)** lotus lectin and **(c)** α-SMA staining are shown as percent area stained per high power field (n=3 mice/group, 15-20 hpf per mouse; for lotus lectin: p=<0.0001 for *NPHS2-rtTA* (SHAM + VEH vs UNx + STZ) and *KLF6^{PODTA}* (SHAM + VEH vs UNx + STZ), p=0.0037 for UNx + STZ (*NPHS2-rtTA* vs *KLF6^{PODTA}*); for α-SMA: p=<0.0001 for *NPHS2-rtTA* (SHAM + VEH vs UNx + STZ) and UNx + STZ (*NPHS2-rtTA* vs *KLF6^{PODTA}*), p=0.0033 for *KLF6^{PODTA}* (SHAM + VEH vs UNx + STZ); **p<0.01, ****p<0.0001; Kruskal-Wallis test with Dunn's post-test). Source data are provided as a Source Data file.

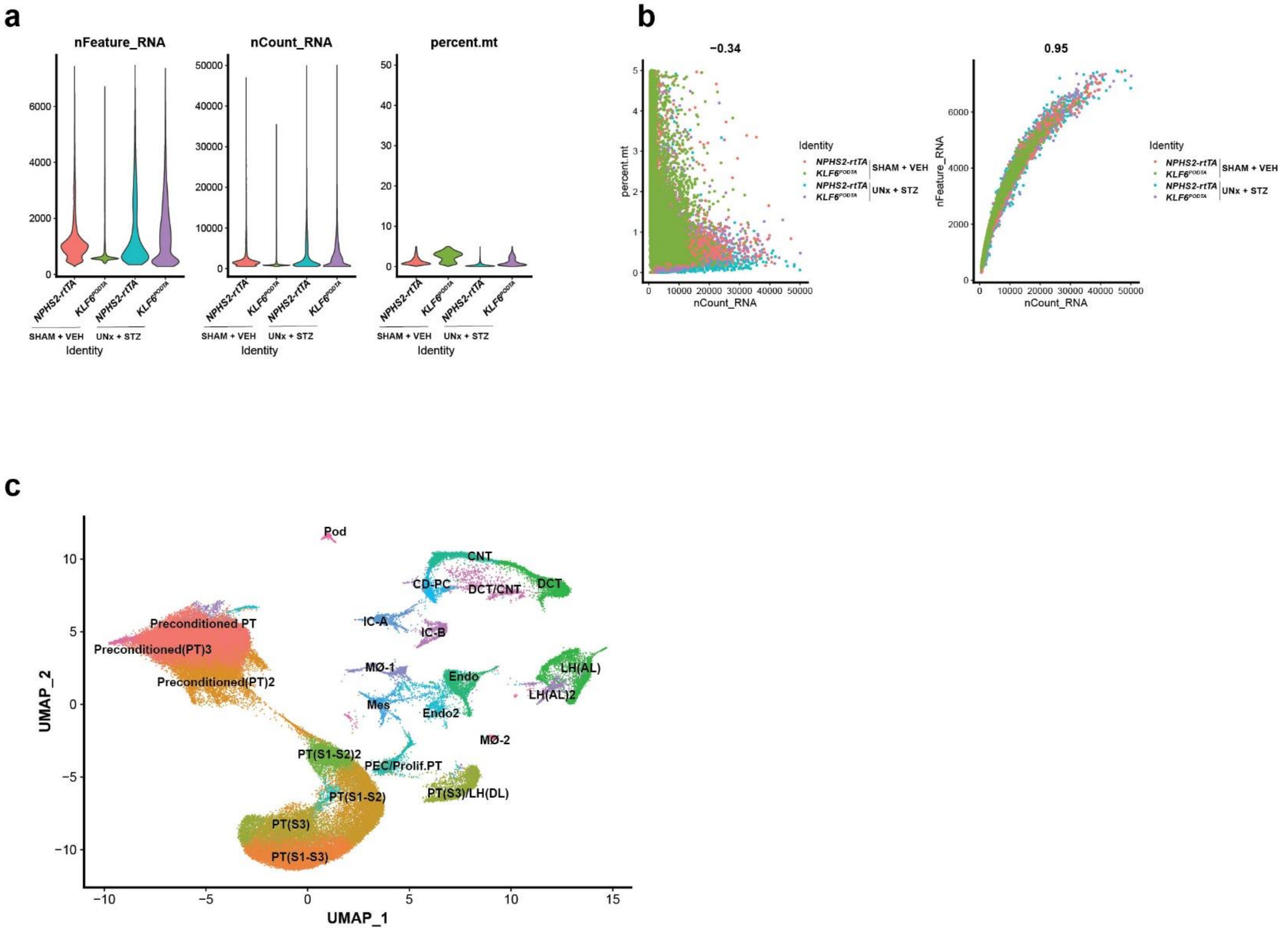

Supplementary Fig. 3. snRNA-seq QC parameters and UMAP plot by unsupervised clustering. (a-b) The number of genes (nFeatures), UMIs (nCount), and percentage of mitochondrial transcripts (percent.mt) are shown as a violin and feature plots. **(c)** Uniform Manifold Approximation and Projection (UMAP) plot illustrates all clusters identified by unsupervised clustering.

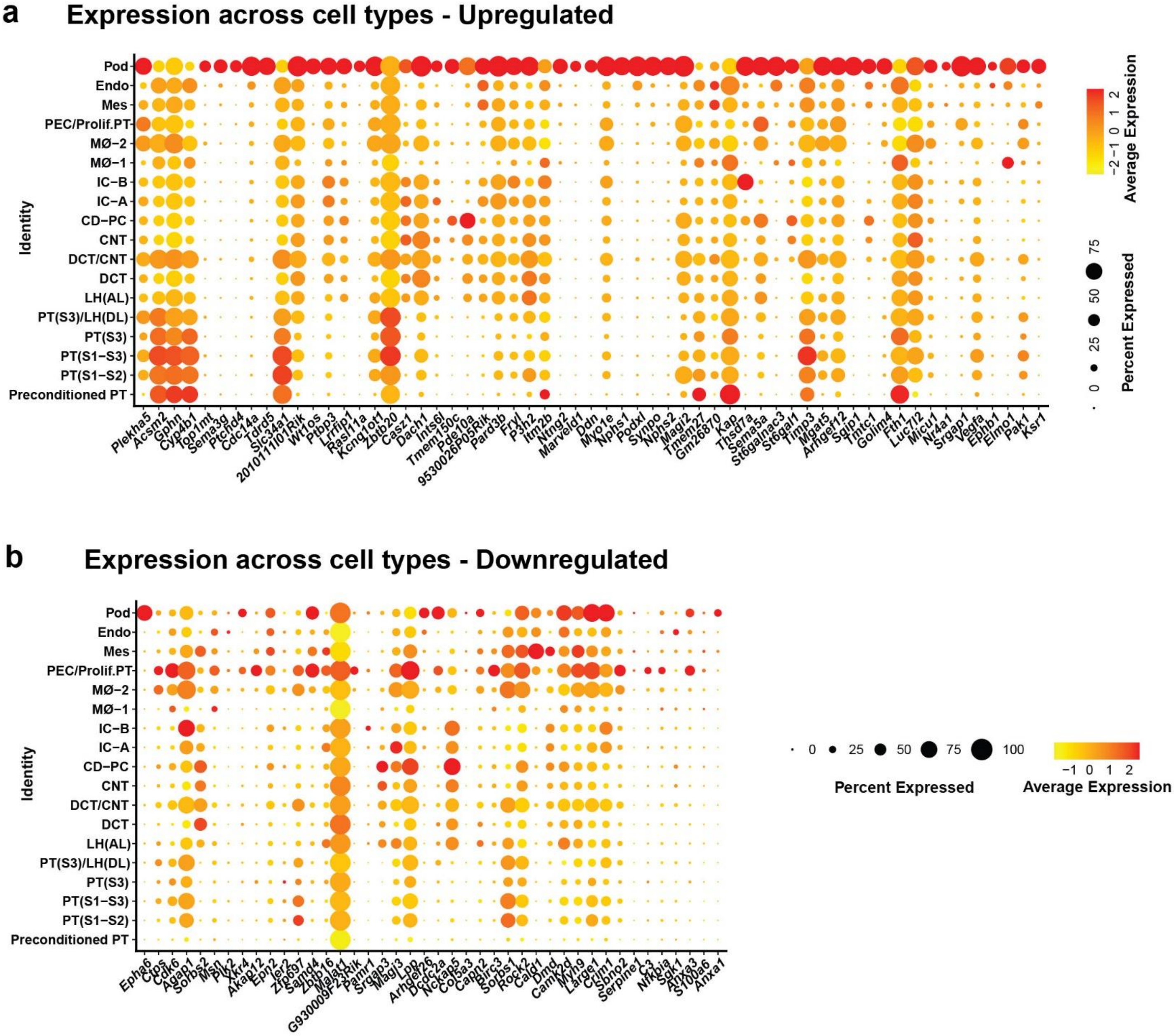

Supplementary Fig. 4. Cell specificity of differentially expressed genes (DEGs) in the podocyte cluster in *KLF6^{PODTA}* vs *NPHS2-rtTA* mice under diabetic conditions. Dot plot of the **(a)** upregulated and **(b)** downregulated genes in *KLF6^{PODTA}* vs *NPHS2-rtTA* mice under diabetic conditions across all cell clusters.

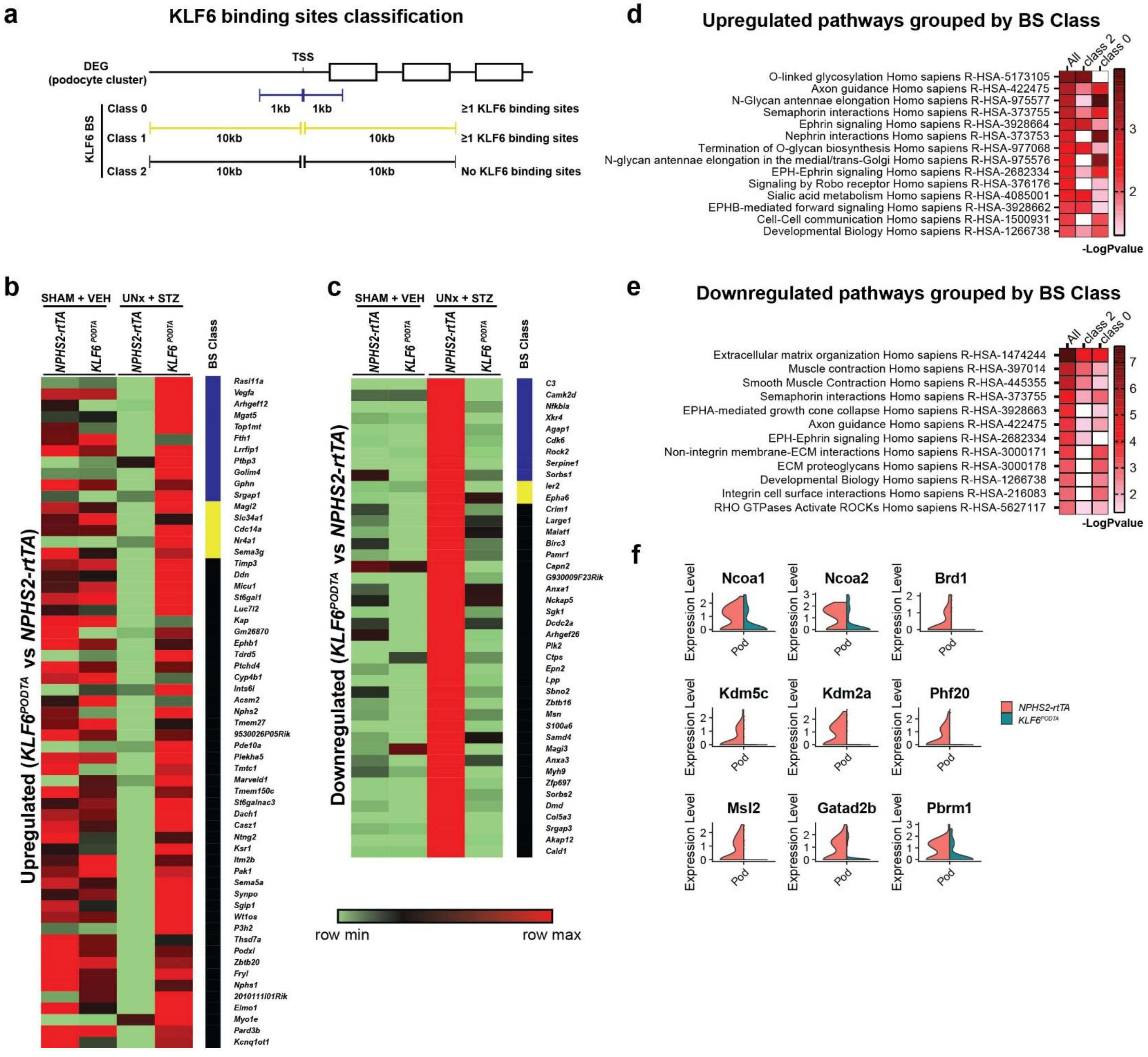

Supplementary Fig. 5. ChIP enrichment analysis for differentially expressed genes with KLF6 binding sites. (a) Schematic illustrating KLF6 binding site classification: class 0 with ≥1 KLF6 binding site within ±1 kb of TSS; class 1 with ≥1 KLF6 binding site at ±1 to 10 kb from TSS; and class 2 with no KLF6 binding site within ± 10 kb from TSS. **(b)** Heatmap showing expression of the upregulated differentially expressed genes (DEGs) in the podocyte cluster, grouped by the KLF6 binding site class. **(c)** Heatmap of the downregulated DEGs in the podocyte cluster, grouped by the KLF6 binding site class. Pathway enrichment analysis of **(d)** upregulated and

(e) downregulated genes based on the KLF6 binding site class. **(f)** Violin plots of differentially expressed chromatin organization related genes in the podocyte cluster.

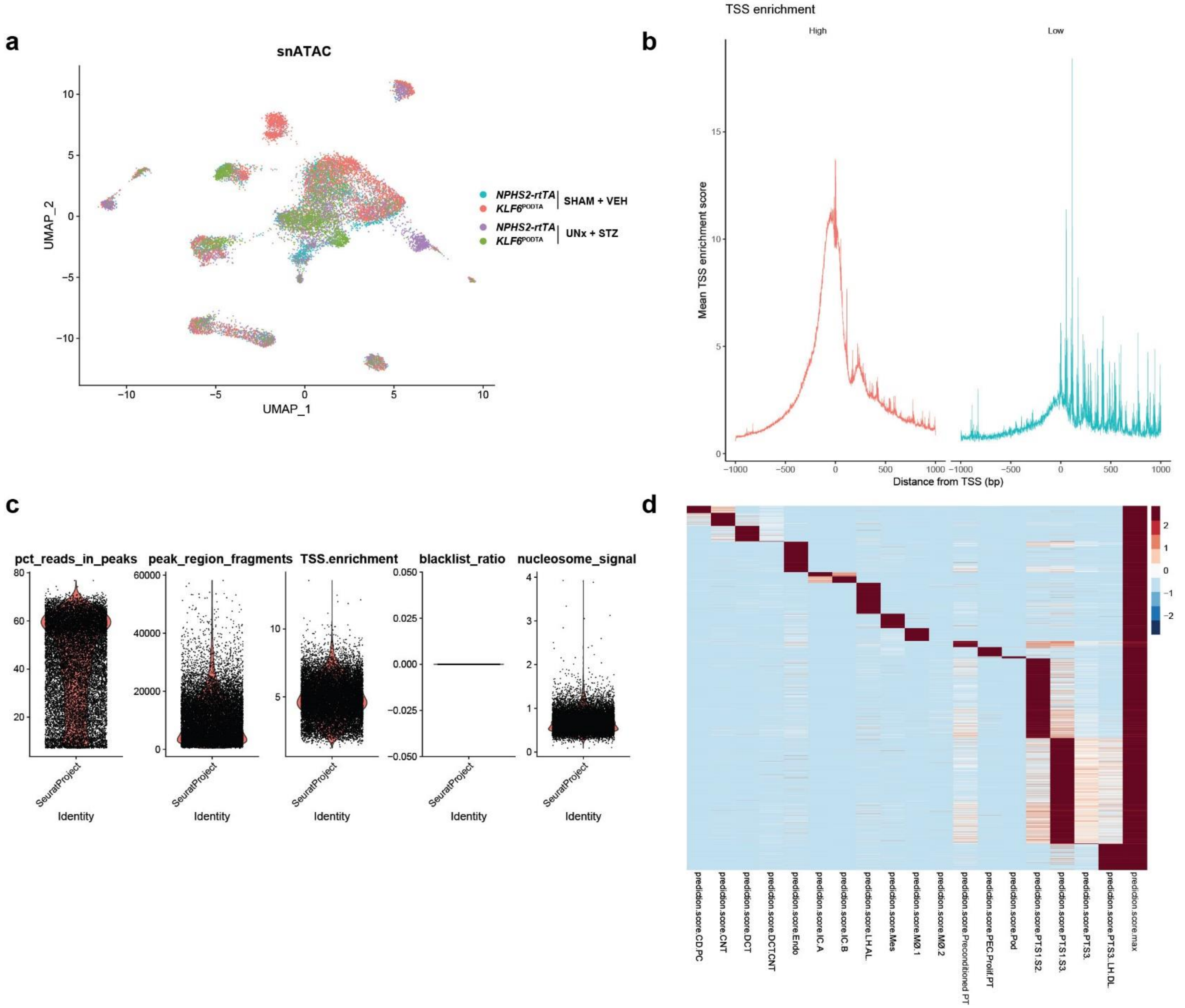

Supplementary Fig. 6. snATAC-seq QC parameters and UMAP plot by unsupervised clustering. (a) Integrated UMAP showing individual genotypes. **(b)** TSS enrichment score distribution. **(c)** Violin plots showing pct_reads_in_peaks, peak_region_fragments, TSS.enrichment, blacklist_ratio, and nucleosome_signal after QC cutoffs. **(d)** Heat map showing prediction score max for various cell types after successful label transfer from snRNA-seq after data integration with snATAC-seq.

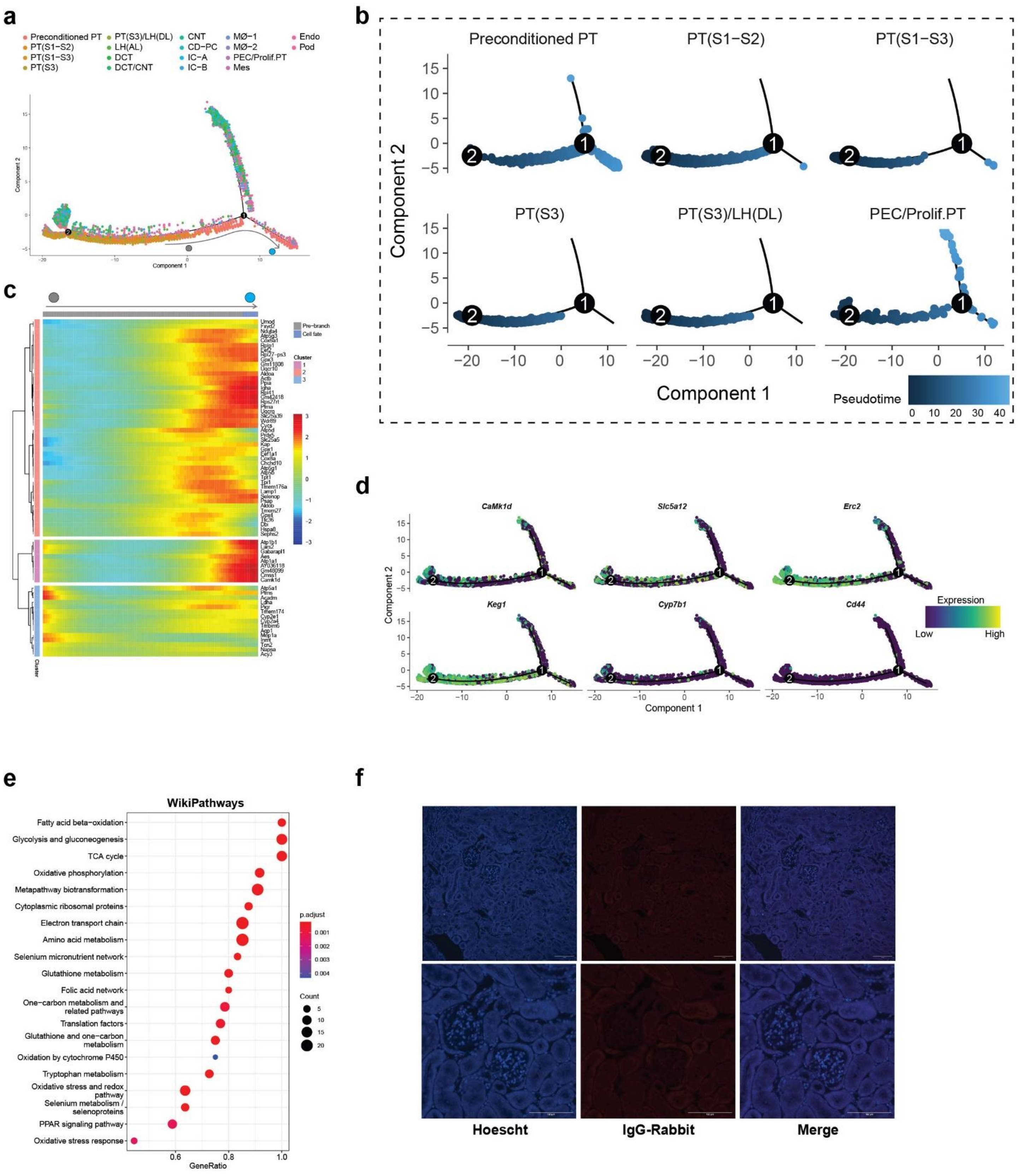

Supplementary Fig. 7. Trajectory analysis utilizing snRNA sequencing data showing the relationship between the preconditioned-PT cluster and other cell clusters in *KLF6*^{PODTA} mice. (a) PCA visualization of

trajectory analysis with pseudotime analysis done with monocle across all cell types (grey circle indicates the horizontal coordinates corresponding to the proximal tubules (PTs) and blue circle indicates the coordinates for Preconditioned-PT). **(b)** PCA visualization of trajectory analysis for individual cell types including preconditioned-PT, PT(S1-S2), PT(S1-S3), PT(S3), PT(S3)/LH(DL), and PEC/Prolif. PT. **(c)** Pseudotemporal expression pattern showing gene expression changes across the horizontal coordinate of the pseudotime analysis from proximal tubules (grey) to preconditioned PT (blue) phenotype. Genes (row) are clustered and cells (column) are ordered according to the pseudotime. **(d)** Expression of key genes for Preconditioned PT (*Camk1d*), PT(S1-S2) (*Slc5a12*), PT(S1-S3) (*Erc2*), PT(S3) (*Keg1*), PT(S3)/LH(DL) (*Cyp7b1*), PEC/Prolif.PT (*Cd44*) along the pseudotime trajectory. **(e)** Pathways enrichment analysis for the genes that change along the pseudotime trajectory of PT cells to Preconditioned-PT. **(f)** Representative images of controls for immunostaining of Camk1d (Scale bar =100 µm).

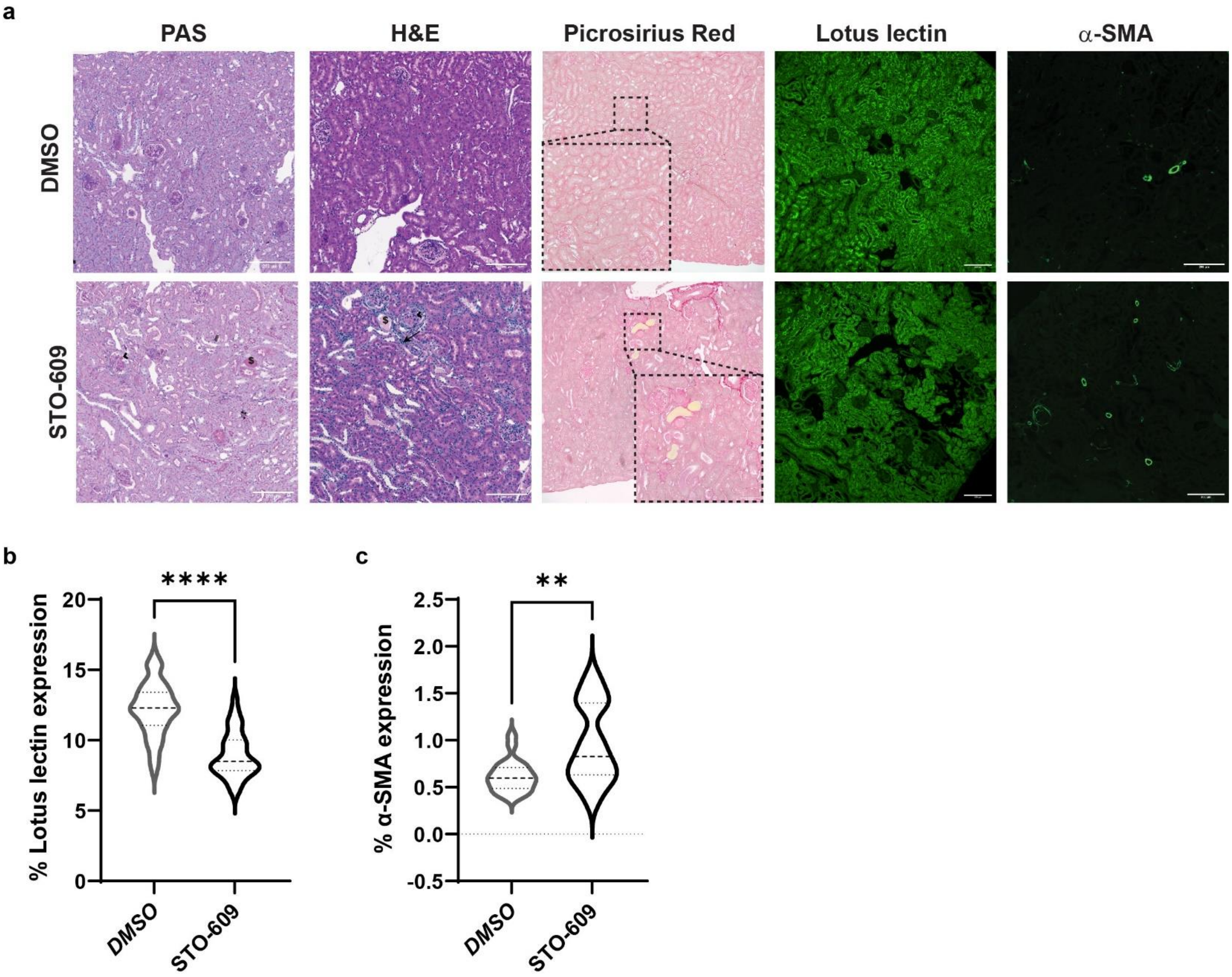

Supplementary Fig. 8. Increase in tubular injury in the diabetic *KLF6*^{PODTA} mice treated with STO-609. (a) Representative image of PAS, H&E, picrosirius red staining (with inset), lotus lectin staining, and α-SMA immunostaining. Black arrowheads indicate sclerotic glomeruli; $ indicates protein casts; and black arrows indicate areas of interstitial inflammation (Scale bar =200 µm and 100 µm for inset). Quantification of **(b)** lotus lectin and **(c)** α-SMA staining are shown as percent are stained per high power field (n=3 mice/group, 15-20 hpf per mouse; p=<0.0001 for lotus lectin, p=0.0013 for α-SMA; **p<0.01, ****p<0.0001; Kruskal-Wallis test with Dunn's post-test). Source data are provided as a Source Data file.

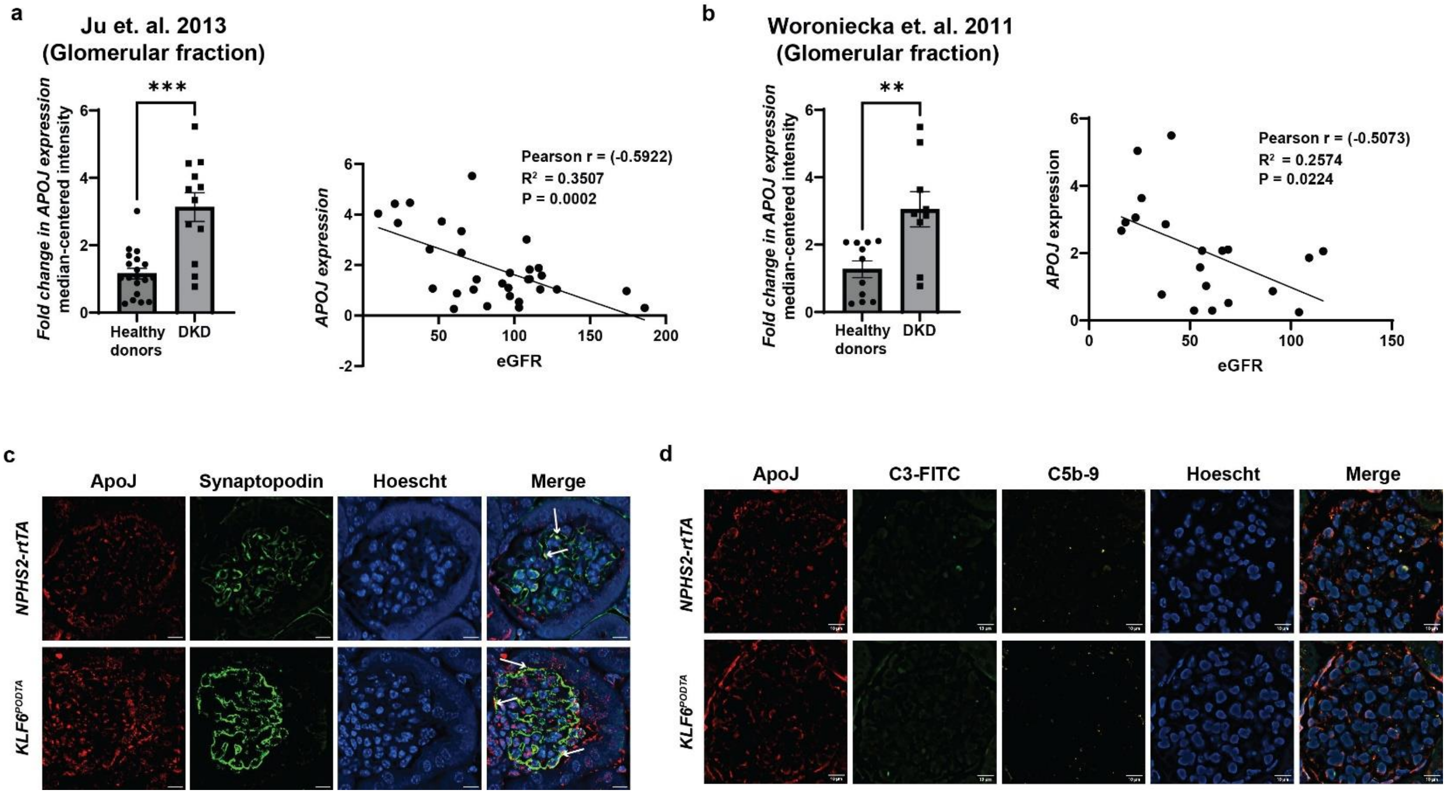

Supplementary Fig. 9. Glomerular and podocyte ApoJ expression in diabetic kidney disease. (a-b) To examine the *APOJ* expression, we used previously reported gene expression arrays from Ju et al., and Woroniecka et al. collected from microdissected glomeruli of kidney biopsy specimens with DKD and healthy living donor biopsy specimens and analysis of correlation with GFR (n=9-21, p=0.0004 (Ju et al.) and p=0.0042 (Woroniecka et al.), **p<0.01,***p<0.001, Mann-Whitney test, data presented as mean±SEM). **(c)** Representative image of co-immunostaining of synaptopodin with ApoJ. White arrows indicate the clusterin co-localization with podocyte markers (Scale bar=10 µm). **(d)** Representative image of co-immunostaining of immune makers such as C3-FITC and C5b-9 with ApoJ (Scale bar=10 µm). Source data are provided as a Source Data file.

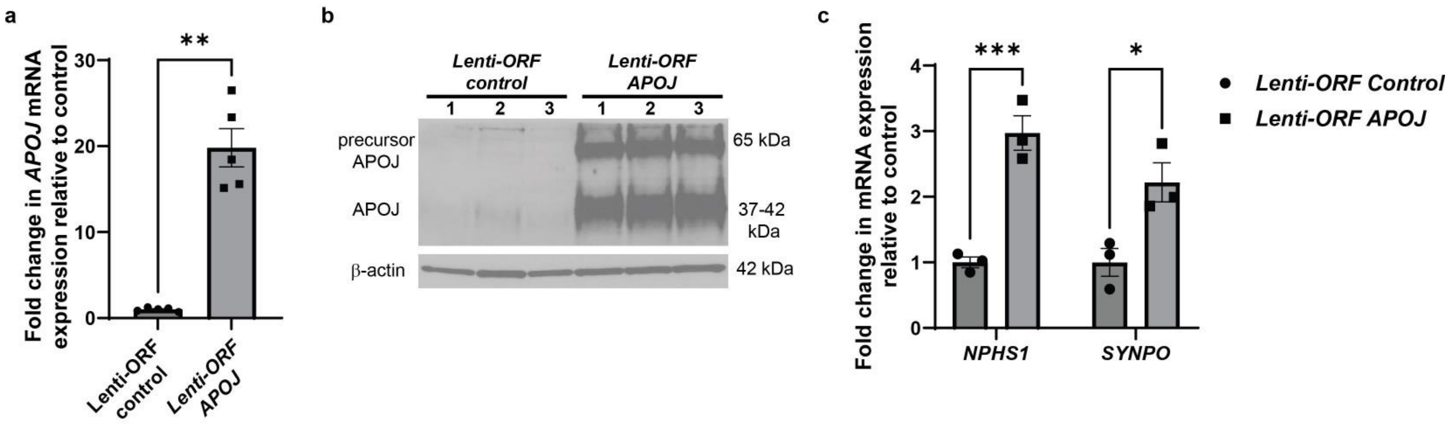

Supplementary Fig. 10. *APOJ* overexpression in cultured human podocytes. (a) Fold change in *APOJ* mRNA expression in *Lenti-ORF-APOJ* relative to *Lenti-ORF-control* (n=5, p=0.0079; **p<0.01, Mann-Whitney test, data presented as mean±SEM) **(b)** Western blot for ApoJ and β-actin **(c)** mRNA expression of podocyte markers (*NPHS1* and *SYNPO*) in *Lenti-ORF-APOJ* relative to *Lenti-ORF-control* (n=3, p=0.0006 for *NPHS1* and p=0.0108 for *SYNPO*; *p<0.05, ***p<0.001, Mann-Whitney test, data presented as mean±SEM). Source data are provided as a Source Data file.

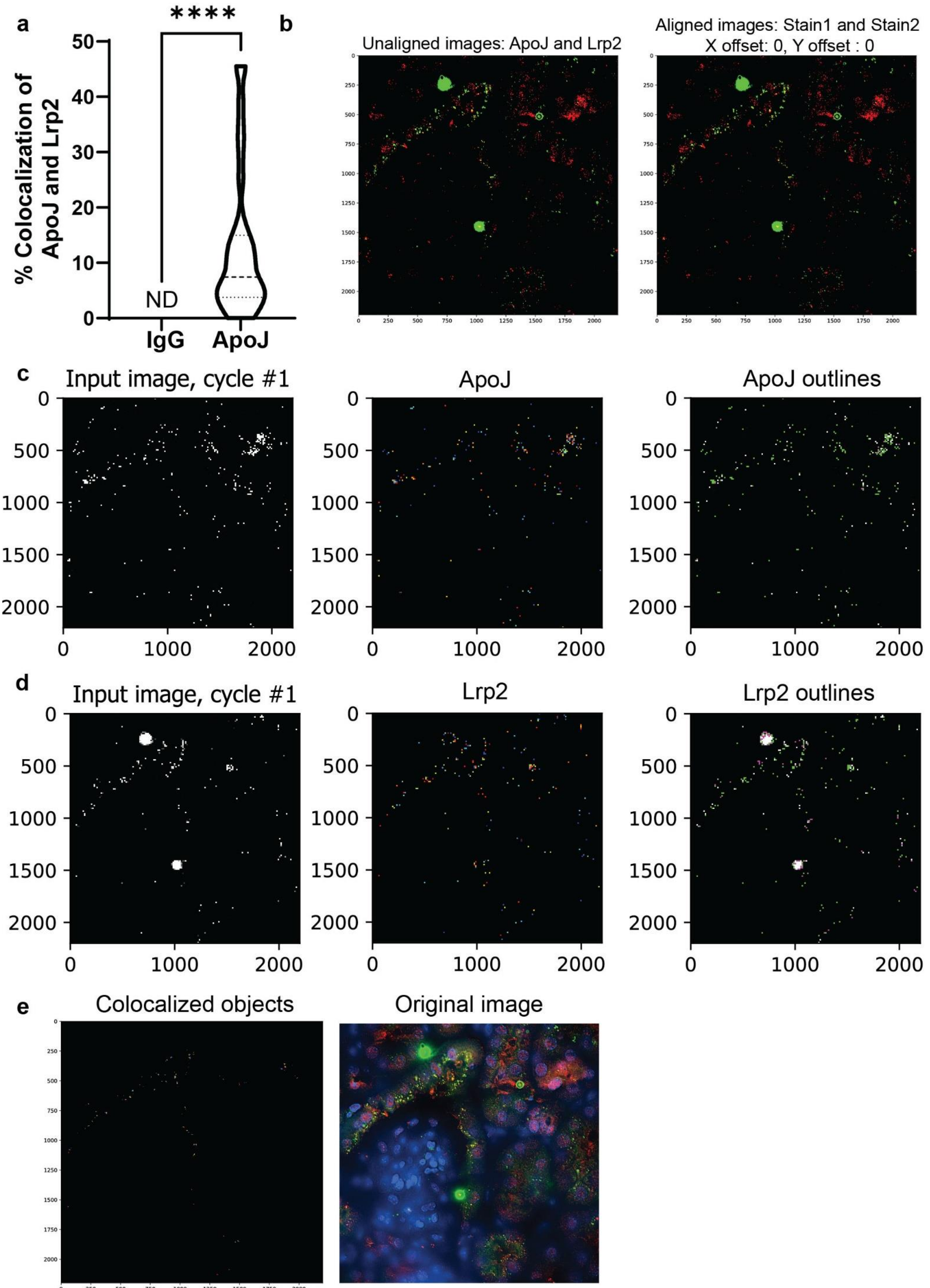
a
% Colocalization of ApoJ and Lrp2
****
ND
IgG
ApoJ
b
Unaligned images: ApoJ and Lrp2
Aligned images: Stain1 and Stain2
X offset: 0, Y offset : 0
c
Input image, cycle #1
ApoJ
ApoJ outlines
d
Input image, cycle #1
Lrp2
Lrp2 outlines
e
Colocalized objects
Original image

Supplementary Fig. 11. Quantification of colocalization of ApoJ and Lrp2. (a) % colocalized objects between ApoJ and Lrp2 compared to Lrp2, (ND: not detected, p=<0.0001; ****p<0.0001, Mann-Whitney test, n=3 mice/group, 10 representative images/mice) **(b)** Alignment of the ApoJ and Lrp2 channels in CellProfiler. **(c-d)** Identification of primary objects for ApoJ and Lrp2 channel. **(e)** Mask indicating the colocalized objects between ApoJ and Lrp2. Source data provided as a Source Data file.
