# Supplementary material for: Podocyte-specific KLF6 primes proximal tubule CaMK1D signaling to attenuate diabetic kidney disease
Source: Nat Commun. 2024 Sep 13;15:8038. doi: 10.1038/s41467-024-52306-5 (PMC11399446; doi:10.1038/s41467-024-52306-5)
Supplement: Supplementary file 3 — Description of additional supplementary files [file 41467_2024_52306_MOESM3_ESM.docx]

**DESCRIPTION OF ADDITIONAL SUPPLEMENTARY FILES**

**Supplementary Data 1:** Nuclei counts per cluster from snRNA-seq

**Supplementary Data 2:** Differentially expressed genes per cluster from snRNA-seq

**Supplementary Data 3:** Differentially accessible chromatin region per cluster from snATAC-seq

**Supplementary Data 4:** Differentially transcription factor motifs per cluster from snATAC-seq

**Supplementary Data 5:** Identified and differentially abundant proteins in podocyte conditioned media and urine
