# Supplementary material for: Podocyte-specific KLF6 primes proximal tubule CaMK1D signaling to attenuate diabetic kidney disease
Source: Nat Commun. 2024 Sep 13;15:8038. doi: 10.1038/s41467-024-52306-5 (PMC11399446; doi:10.1038/s41467-024-52306-5)
Supplement: Supplementary file 9 — Reporting Summary [file 41467_2024_52306_MOESM9_ESM.pdf]

nature portfolio

Corresponding author(s): Sandeep Mallipattu

Last updated by author(s): 2024/08/02

Reporting Summary

Nature Portfolio wishes to improve the reproducibility of the work that we publish. This form provides structure for consistency and transparency in reporting. For further information on Nature Portfolio policies, see our Editorial Policies and the Editorial Policy Checklist.

Statistics

For all statistical analyses, confirm that the following items are present in the figure legend, table legend, main text, or Methods section.

n/a	Confirmed	
☐	☒	The exact sample size (n) for each experimental group/condition, given as a discrete number and unit of measurement
☐	☒	A statement on whether measurements were taken from distinct samples or whether the same sample was measured repeatedly
☐	☒	The statistical test(s) used AND whether they are one- or two-sided<br>*Only common tests should be described solely by name; describe more complex techniques in the Methods section.*
☒	☐	A description of all covariates tested
☐	☒	A description of any assumptions or corrections, such as tests of normality and adjustment for multiple comparisons
☐	☒	A full description of the statistical parameters including central tendency (e.g. means) or other basic estimates (e.g. regression coefficient) AND variation (e.g. standard deviation) or associated estimates of uncertainty (e.g. confidence intervals)
☐	☒	For null hypothesis testing, the test statistic (e.g. F, t, r) with confidence intervals, effect sizes, degrees of freedom and P value noted<br>*Give P values as exact values whenever suitable.*
☒	☐	For Bayesian analysis, information on the choice of priors and Markov chain Monte Carlo settings
☒	☐	For hierarchical and complex designs, identification of the appropriate level for tests and full reporting of outcomes
☐	☒	Estimates of effect sizes (e.g. Cohen's d, Pearson's r), indicating how they were calculated

Our web collection on statistics for biologists contains articles on many of the points above.

Software and code

Policy information about availability of computer code

Data collection	10X chromium systems,NovaSeqS4, ReproSil C-18 (3 uM particle) column, Q Exactive HF Hybrid Quadrupole-Orbitrap Mass Spectrometer (Thermo Fisher Scientific) or TripleTOF 5600+ (Sciex), Nikon Eclipse i90 microscope and DS-Qi1Mc camera
Data analysis	CellRanger 3.1.0-v2 ; Cell Ranger atac-2.0.0; SeaWulf-the HPC Cluster at Stony Brook University; R-packages: Seurat 4.3.0, Signac 1.8.0, clusterprofiler 4.6.2, Monocle-v2, edgebundler 0.1.4; ArchR 1.0.2; Enrichr libraries of KEGG 2019 (mouse) pathways, WikiPathways 2019 (mouse), and Reactome pathways; Morpheus software; Thermo Fisher Proteome Discoverer v2.4; Scaffold; Nephroseq; ImageJ (NIH); CellProfiler; Excel; GraphPad Prism 9.0

For manuscripts utilizing custom algorithms or software that are central to the research but not yet described in published literature, software must be made available to editors and reviewers. We strongly encourage code deposition in a community repository (e.g. GitHub). See the Nature Portfolio guidelines for submitting code & software for further information.

Data

Policy information about availability of data

All manuscripts must include a data availability statement. This statement should provide the following information, where applicable:

- Accession codes, unique identifiers, or web links for publicly available datasets
- A description of any restrictions on data availability
- For clinical datasets or third party data, please ensure that the statement adheres to our policy

All data needed to evaluate the conclusions in the paper are present in the paper and/or the Supplemental Materials. Source data are provided with this paper. All

raw data from snRNA-seq and ATAC-seq have been deposited in the Gene Expression Omnibus GSE171854 [https://www.ncbi.nlm.nih.gov/geo/query/acc.cgi?acc=GSE171722] and GSE230681 [https://www.ncbi.nlm.nih.gov/geo/query/acc.cgi?acc=GSE230681. Raw data from proteomics have been deposited in Massive Database (accession no. MSV000092123, reviewer's password: KLF6OE2023).

CODE AVAILABILITY
R code and Seurat RDS data object are available through the following link: https://github.com/MallipattuLab/KLF6_PODTA

Research involving human participants, their data, or biological material

Policy information about studies with human participants or human data. See also policy information about sex, gender (identity/presentation), and sexual orientation and race, ethnicity and racism.

Reporting on sex and gender	De-identified (archived, with no linking clinical/demographic information) FPPE human samples were used for staining and previously reported Nephroseq data from Ju et.al. and Woroniecka et. al. was interrogated. Sex and gender information were not disclosed
Reporting on race, ethnicity, or other socially relevant groupings	Race, ethnicity or other socially relelvant information were not disclosed since we are utilizing previously deposited (de-identified" data).
Population characteristics	"See above"
Recruitment	Previously deposited (archived) de-identified samples and data were used for the study
Ethics oversight	The study was approved by the Stony Brook University Institutional Review Board

Note that full information on the approval of the study protocol must also be provided in the manuscript.

Field-specific reporting

Please select the one below that is the best fit for your research. If you are not sure, read the appropriate sections before making your selection.

[x] Life sciences [ ] Behavioural & social sciences [ ] Ecological, evolutionary & environmental sciences

For a reference copy of the document with all sections, see nature.com/documents/nr-reporting-summary-flat.pdf

Life sciences study design

All studies must disclose on these points even when the disclosure is negative.

Sample size	Using a standard deviation of 20% change in effect size (specific for the experiment, i.e., animals, HG/NG, diabetic sera) from the preliminary data and accounting for a possible 10% loss in sample size (i.e., mortality in animals, specimen loss)
Data exclusions	No data were excluded except for samples <1% were excluded from experiments due to issues with sample collections or outliers
Replication	At least 3 biological replicates were used to ensure the reproducibility of the experimental findings
Randomization	Animals were randomly assigned to their experimental groups based on their genotypes
Blinding	To eliminate bias, during the analysis of specific experiments and quantifications, the individual performing the analysis were blinded to mouse genotypes and experimental groups.

Reporting for specific materials, systems and methods

We require information from authors about some types of materials, experimental systems and methods used in many studies. Here, indicate whether each material, system or method listed is relevant to your study. If you are not sure if a list item applies to your research, read the appropriate section before selecting a response.

Materials & experimental systems

n/a	Involved in the study
	x Antibodies
	x Eukaryotic cell lines
x	Palaeontology and archaeology
	x Animals and other organisms
x	Clinical data
x	Dual use research of concern
x	Plants

Methods

n/a	Involved in the study
x	ChIP-seq
	x Flow cytometry
x	MRI-based neuroimaging

Antibodies

Antibodies used	mouse anti-WT1 (Santa Cruz, sc-7385, 1:50 dilution), goat anti-synaptopodin (Santa Cruz, sc21537, 1:200 dilution), rabbit anti-KLF6 (Santa Cruz, AP6588B, 1:150 dilution), mouse anti-α-SMA (Sigma-Aldrich, A1978, 1:10,000 dilution), rabbit anti-CaMK1D (Invitrogen, PA5-21957, 1:100 dilution), goat anti-ApoJ (Novus Biologicals, NBP1-06027, 1:100 dilution for IF), mouse anti-Lrp2 (Novus Biologicals, NB110-96417, 1:100 dilution), rabbit anti-TOM20 (Abcam, ab78547, 1:100 dilution), Fluorescein-conjugated goat IgG to mouse complement C3 (MP Biomedicals, 085500, 1:100 dilution), mouse anti-C5b-9 (Santa Cruz, sc-66190, 1:100 dilution), fluorophore-linked secondary antibody (Alexa Fluor 647 Donkey anti-mouse, Fluor 488 Goat anti-rabbit, or Fluor 568 Donkey anti-rabbit from Life Technologies, 1:300 dilution), rabbit anti-DRP1 (Invitrogen, MA5-26255, 1:1000 dilution), rabbit anti-phospho-DRP1(Ser637) (Invitrogen, PA5-101038, 1:1000 dilution), goat anti-ApoJ (R&D, AF2747, 1:100 dilution for IHC and 1:1000 for western), anti-goat horseradish peroxidase secondary (Sigma Aldrich, AP200P, 1:300 dilution), rabbit anti-ApoJ (Cell Signaling technology, 42143, 1:1000 dilution), mouse anti-β-actin (Sigma-Aldrich, A1978, 1:5000 dilution), fluorescein-labeled lotus lectin (Vector Labs, 1:100 dilution) and/or Hoechst (Invitrogen, 1:1000 dilution)
Validation	Validation of all the antibodies used in the study is available on manufacture's website. IgG control was also used for validation of all antibodies used for immunostaining.

Eukaryotic cell lines

Policy information about cell lines and Sex and Gender in Research

Cell line source(s)	HK2 cells (CRL-2190) were purchased from ATCC, Immortalized human podocytes were provided by Dr. Moin Saleem (University of Bristol, Southmead Hospital, Bristol, UK) , male primary mouse podocytes, male primary mouse PT cells were used for the study
Authentication	GFP-labelled primary mouse podocytes were obtained using FACS sorting. Other cell lines were validated in previous publications or by manufacturer's website
Mycoplasma contamination	No mycoplasma contamination was found in the HK2 and the immortalized human podocyte cell lines
Commonly misidentified lines (See ICLAC register)	N/A

Animals and other research organisms

Policy information about studies involving animals; ARRIVE guidelines recommended for reporting animal research, and Sex and Gender in Research

Laboratory animals	FVB/N mice with podocyte specific overexpression of KLF6, and NPHS2-rtta mice within the age range of 16-20 weeks were used in this study and C57BL/6 mice within the age range of 5-6 weeks were used for primary mouse PT cells isolation. The mice were housed in our animal facility with free access to food and water and 12 hours day/light cycle, in ambient temperature and humidity conditions
Wild animals	N/A
Reporting on sex	Only male mice were used for this study
Field-collected samples	N/A
Ethics oversight	All animal studies conducted were approved by the Stony Brook University Animal Institute Committee. The National Institutes of Health Guide for the Care and Use of Laboratory Animals was followed strictly.

Note that full information on the approval of the study protocol must also be provided in the manuscript.

Plants

Seed stocks

Report on the source of all seed stocks or other plant material used. If applicable, state the seed stock centre and catalogue number. If plant specimens were collected from the field, describe the collection location, date and sampling procedures.

Novel plant genotypes

Describe the methods by which all novel plant genotypes were produced. This includes those generated by transgenic approaches, gene editing, chemical/radiation-based mutagenesis and hybridization. For transgenic lines, describe the transformation method, the number of independent lines analyzed and the generation upon which experiments were performed. For gene-edited lines, describe the editor used, the endogenous sequence targeted for editing, the targeting guide RNA sequence (if applicable) and how the editor was applied.

Authentication

Describe any authentication procedures for each seed stock used or novel genotype generated. Describe any experiments used to assess the effect of a mutation and, where applicable, how potential secondary effects (e.g. second site T-DNA insertions, mosiacism, off-target gene editing) were examined.

Flow Cytometry

Plots

Confirm that:

☐ The axis labels state the marker and fluorochrome used (e.g. CD4-FITC).

☐ The axis scales are clearly visible. Include numbers along axes only for bottom left plot of group (a 'group' is an analysis of identical markers).

☐ All plots are contour plots with outliers or pseudocolor plots.

☒ A numerical value for number of cells or percentage (with statistics) is provided.

Methodology

Sample preparation

In order to obtain primary mouse podocytes from GFP-labeled KLF6PODTA and NPHS2-rtTA mice, the mice were perfused using sterile PBS with dynabeads and the glomeruli was isolated as previously reported. Glomerular cell digestion was carried out using collagenase, single cell suspension was obtained and GFP-labelled podocytes were isolated using FACS.
For measurement of mitochondrial membrane potential: Cells were trypsinized, washed with PBS, and incubated with 1,1′,3,3,3′,3′-hexamethylindodicarbo-cyanine iodide (DilC1) alone or with DilC1 and carbonyl cyanide 3-chlorophenylhydrazone (CCCP) and the difference in fluorescence intensity was measured between the groups using FACS.

Instrument

FACSaria IIIu and cytoflex LX

Software

The software for FaCSaria IIIu was FACSdiva and for Cytoflex LX was Cytexpert

Cell population abundance

20000 cells per sample were collected

Gating strategy

GFP-labelled cells were collected to culture primary mouse podocytes and for DilC1 and CCCP red excitation and far red emission was used as recommended by manufacturer's protocol

☐ Tick this box to confirm that a figure exemplifying the gating strategy is provided in the Supplementary Information.
